# Supplementary material for: Non-Destructive Monitoring via Electrochemical NADH Detection in Murine Cells
Source: Biosensors (Basel). 2022 Feb 10;12(2):107. doi: 10.3390/bios12020107 (PMC8869533; doi:10.3390/bios12020107)
Supplement: Supplementary file 1 [file biosensors-12-00107-s001.zip › biosensors-1564478-supplementary.pdf]

biosensors

Communication

Supplementary Materials: **Non-Destructive Monitoring Via Electrochemical NADH Detection in Murine Cells**

Ju Kyung Lee [1], Han Na Suh [2], Sung Hoon Yoon [2,3], Kyu Hong Lee [2], Sae Young Ahn [4,5], Hyung Jin Kim [6], Sang Hee Kim [1,*]

[1] Department of Medical IT Convergence, Kumoh National Institute of Technology, Gumi 39177, Korea; chejueyes@kumoh.ac.kr
[2] Korea Institute of Toxicology, Jeongeup 56212, Korea; hanna.suh@kitox.re.kr (H.N.S.); seonghoon.yoon@kitox.re.kr (S.H.Y.); khlee@kitox.re.kr (K.H.L.)
[3] Department of Human and Environmental Toxicology, University of Science & Technology, Daejeon 34113, Korea
[4] NDD Inc., Gumi 39253, Korea; sahn@ndd-inc.com
[5] Fuzbien Technology Institute, Rockville, MD 20850, USA
[6] Digital Health Care Research Center, Gumi Electronics and Information Technology Research Institute (GERI), Gumi 39253; hjkim745@geri.re.kr
* Correspondence: shkim@kumoh.ac.kr

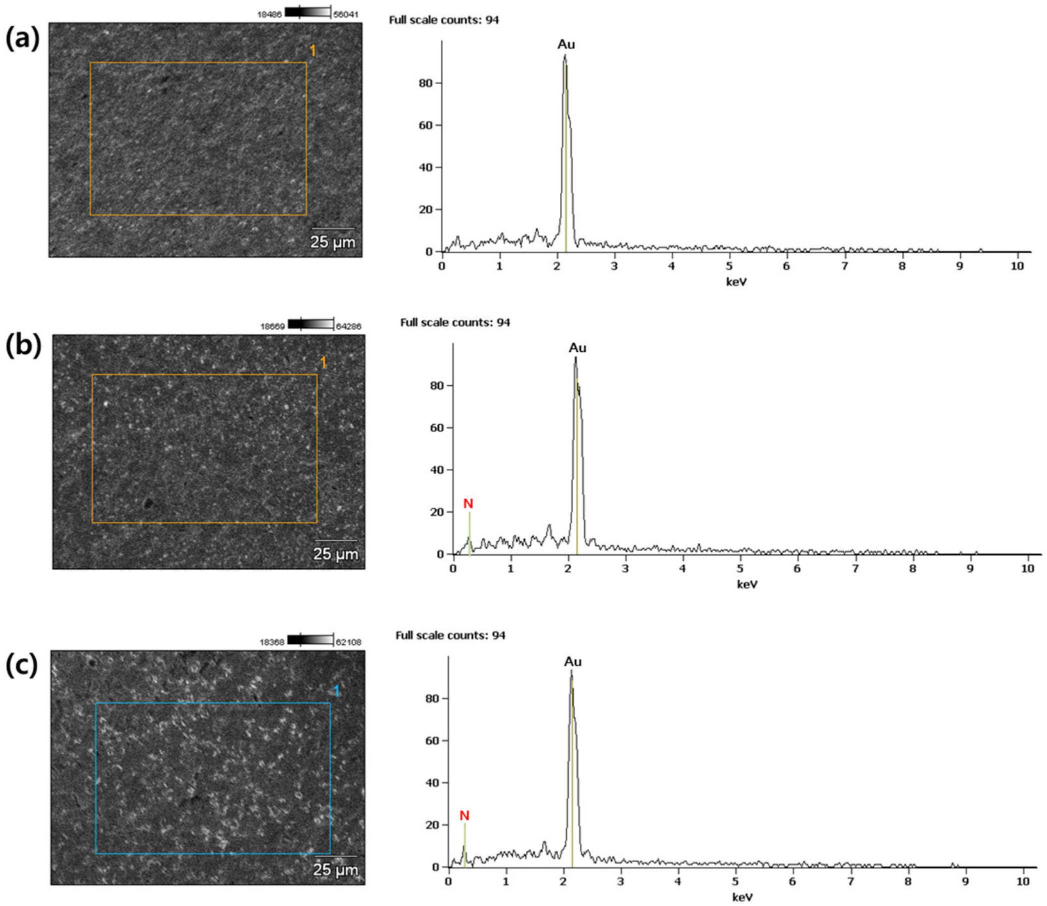

Figure S1. EDAX spectrum of (**a**) bare, (**b**) 4-ATP modified, (**c**) NPQD modified electrode.

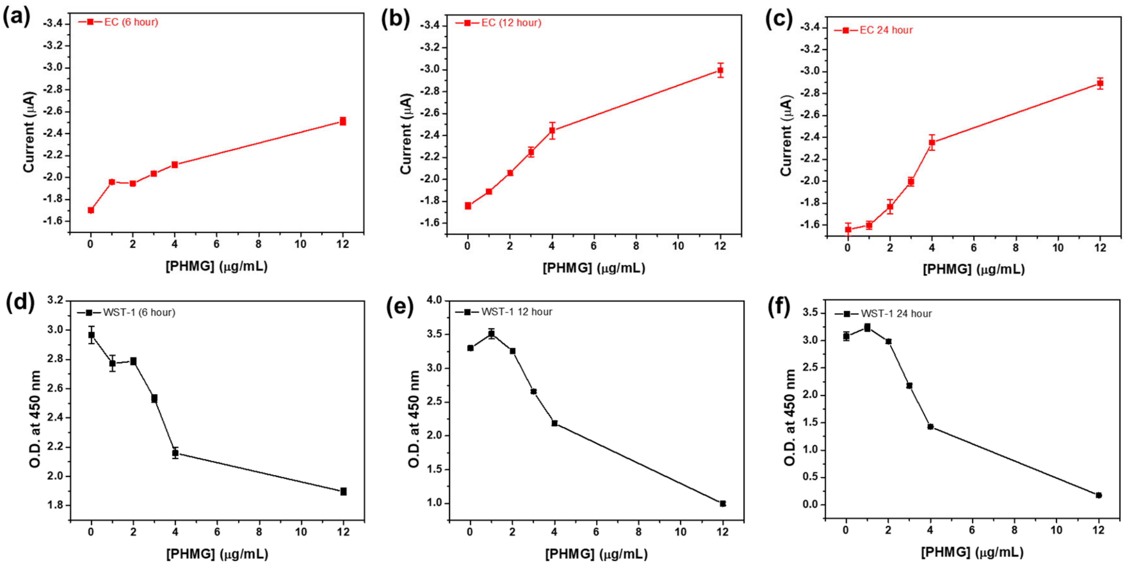

Figure S2. The electrocatalytic (**a**–**c**) and conventional WST-1 (**d**–**f**) sensing data for cell viability monitoring. PHMG was dosed for 6 hours (**a**,**d**), for 12 hours (**b**,**e**), for 24 hours (**c**,**f**).
